# Recurrent Urinary Tract Infections in Older Adults Requiring Hospitalization in an Internal Medicine Ward

**DOI:** 10.3390/microorganisms12112114

**Published:** 2024-10-22

**Authors:** Arturo Artero, Ian López-Cruz, Juan Alberto Aguilera, Laura Piles, Silvia Artero, José María Eiros, Juan Alberola, Manuel Madrazo

**Affiliations:** 1 Doctor Peset University Hospital, Universitat de València, 46017 Valencia, Spain; arturo.artero@uv.es (A.A.); ilopezcruz5@gmail.com (I.L.-C.); juan.alberto.aguilera1994@gmail.com (J.A.A.); laurapilesroger@gmail.com (L.P.); manel.madrazo@gmail.com (M.M.); 2 Gregorio Marañón University Hospital, 28007 Madrid, Spain; silvia.artefull@gmail.com; 3 Rio Hortega University Hospital, Universidad de Valladolid, 47012 Valladolid, Spain; eiros@med.uva.es

**Keywords:** recurrent infections, risk factors, urinary tract infection

## Abstract

Urinary tract infection (UTI) is a common cause of recurrent infections, especially among young women, but also in patients with infections related to the insertion of urological devices. The aim of this study was to determine the recurrent UTI readmission rate among older patients and the risk factors for recurrent UTI in a prospective cohort of patients admitted to the hospital with community-acquired UTI. We assessed the frequency of recurrent UTIs over a one-year follow-up period after discharge and compared the clinical and epidemiological characteristics between cases with and without recurrences. Out of a total of 462 patients included in this study, 35 (7.6%) had a readmission due to UTI. The patients in the overall series had a median age of 78 (69–86) years, and 50% were women. Recurrent UTIs were associated with healthcare-associated UTIs (OR 2.8, 95% CI 1.1–6.9) and *Pseudomonas aeruginosa* infections (OR 2.7, 95% CI 1.1–7.2) according to multivariate analysis. Patients with recurrent UTIs experienced longer hospital stays, with no significant difference in mortality rates. Half of the recurrent UTIs were caused by the same microorganisms as those in primary UTIs, but the prolonged period up to recurrence, with a median of 4 months, suggests that they were mostly reinfections. In conclusion, elderly patients admitted to the hospital with complicated UTIs had a low long-term risk of recurrent UTIs. However, this risk was higher in patients with healthcare-associated infection criteria and in those with *P. aeruginosa* UTIs. Identifying these risk groups may aid in the early detection of recurrent UTIs.

## 1. Introduction

Hospitalized patients with severe infections face a high risk of readmission in the subsequent months [1,2]. Specifically, among patients who survive sepsis and septic shock, the hospital readmission rate within 3 months exceeds 40% [3]. Infection is the most common rehospitalization diagnosis [4], and urinary tract infection (UTI) has been identified as the most common cause of recurrent infections among patients with sepsis [5]. In these cases, many UTIs are acquired in the hospital because of the insertion of urological devices [6].

Conversely, recurrent UTIs are a prevalent issue among sexually active young women, even in the absence of any identifiable structural abnormalities. In these cases, the clinical course is usually benign, hospitalization is generally not necessary, and antibiotic prophylaxis is highly effective, although it carries a risk of developing antibiotic resistance. Recurrent cystitis in women is frequently observed, with previous studies estimating their prevalence to be between 20% and 44% during the first year [7,8,9].

Between these two extremes of UTI severity are patients with complicated community-onset UTIs requiring hospitalization in medical wards. Limited information is available regarding the risk of readmission for new UTI episodes in this situation. Albeit, it has been reported that there is an 8% readmission rate within two months of discharge in patients who survived hospitalization for a complicated UTI [9]. In addition, in older patients, hospitalization for UTIs is more frequent, and there is a higher risk of recurrence due to common comorbidities, such as diabetes mellitus, urinary incontinence, prostatism, or insertion of an indwelling bladder catheter [10,11,12]. 

Recurrent UTIs present a significant healthcare challenge, particularly among the older population. The results of studies on recurrent urinary tract infections conducted in different contexts—such as young women or patients with sepsis admitted to intensive care units—may not be applicable to elderly patients with complicated UTIs admitted to medical wards. Understanding the readmission rates and identifying the risk factors can help in developing targeted interventions to reduce recurrence and improve patient outcomes. This study aims to determine the readmission rate for recurrent urinary tract infections in older patients and identify the associated risk factors based on a prospective cohort of individuals hospitalized with community-acquired UTI.

## 2. Material and Methods

### 2.1. Study Design and Patients

This prospective observational study enrolled patients aged 65 and above, admitted to a university hospital with a diagnosis of community-acquired UTI between January 2016 and December 2021. The initial UTI diagnosis was made in the emergency department and confirmed by the attending physician upon admission. Each case underwent meticulous review by the authors, involving comprehensive examination of medical history. Laboratory and microbiological diagnostic procedures were reviewed. Cases presenting with clinical syndromes indicative of other conditions or classified as asymptomatic bacteriuria were excluded. Additional exclusion criteria included nosocomial infections (UTIs occurring 72 h after hospital admission), cases transferred from the intensive care unit (ICU), patients that did not survive the initial episode of hospitalization, and patients who refused to sign the informed consent to participate in this study.

### 2.2. Definitions

Patients were grouped according to the readmission pattern into two categories: short-term readmission and long-term readmission. Short-term readmission was defined as readmission within 90 days after discharge. Long-term readmission was defined as readmission occurring between 3 and 12 months after discharge. SOFA and qSOFA scores were measured upon the patient’s presentation to the emergency department (ED). In this study, acute UTIs were classified as complicated if the patient presented with structural or functional abnormalities of the urinary tract, suggesting a high risk of treatment failure and the potential for significant complications [13]. Asymptomatic bacteriuria was defined as the isolation of a specified quantitative count of bacteria in an appropriately collected urine specimen from an individual without symptoms of a UTI [14].

The following comorbidities were recorded after reviewing the electronic medical history and following a protocol [15] with the diagnostic criteria used in clinical practice: cognitive impairment, diabetes mellitus, chronic kidney disease, chronic obstructive pulmonary disease, and chronic liver disease.

The Charlson Comorbidity Index was utilized to evaluate the total burden of comorbidities, with a score of 3 or higher indicating significant comorbidity [16].

Clinical data were obtained via patient interviews and physical examinations. Fever was defined as either a temperature of ≥38 °C reported by the patient at home or a measured temperature of ≥38 °C in the emergency department. Laboratory assessments comprised complete blood counts, coagulation tests, and a comprehensive blood chemistry analysis, assessing liver and renal function, electrolyte levels, procalcitonin, and C-reactive protein levels.

Sepsis was classified according to the Sepsis-3 criteria, which define it as an acute increase of ≥2 points in the total SOFA score attributable to infection. Both the SOFA and quick SOFA (qSOFA) scales were used in accordance with their original definitions. The Acute Physiology and Chronic Health Evaluation II (APACHE II) score was employed to assess illness severity at admission. The Barthel Index was used to evaluate the functional status of patients by measuring their level of independence in activities of daily living [17,18].

Inadequate empirical antimicrobial therapy (IEAT) was defined as cases where an infection was not effectively treated at the time the causative microorganism and its antimicrobial susceptibility were identified. This included two scenarios: the absence of antimicrobial agents effective against a specific class of microorganisms and the administration of an antimicrobial agent to which the causative microorganism was resistant [19].

### 2.3. Microbiological Studies

Microbiological information was obtained through urine and blood cultures, including susceptibility testing. This procedure included the identification of bacteremia, determination of the causative agents of urinary tract infections (UTIs) via culture isolation, evaluation of resistance patterns demonstrated by the isolated microorganisms, and detection of cases involving polymicrobial infections. Microorganisms from positive blood cultures were identified using a Bruker MALDI Biotyper system (Beckman Coulter, Brea, CA, USA). For antimicrobial susceptibility testing, two automated systems were utilized: a DxM MicroScan WalkAway microbiology system (Beckman Coulter, Brea, CA, USA) and a VITEK 2 system (bioMérieux, Inc., Hazelwood, MO, USA). These systems employed microbroth dilution techniques, adhering to a combination of guidelines established by the CLSI (https://clsi.org/, accessed on 15 October 2024) and the EUCAST (www.eucast.org, accessed on 15 October 2024). Blood samples were drawn in the emergency department and processed using a BacT/ALERT^®^ VIRTUO™ system (bioMérieux Inc., Hazelwood, MO, USA), an automated platform for blood culture and microorganism detection. When positive blood cultures were identified, microorganisms were analyzed with a Bruker MALDI Biotyper system (Beckman Coulter, Brea, CA, USA). To assess drug sensitivity and resistance, a DxM MicroScan WalkAway system (Beckman Coulter, Brea, CA, USA) and a VITEK 2 system (bioMérieux, Inc., Hazelwood, MO, USA) were employed. These systems applied microbroth dilution methods, adhering to the standards set by both the CLSI and the EUCAST.

Multidrug resistance (MDR) was defined following the international expert guidelines established by Magiorakos et al. [20] as the lack of susceptibility to at least one agent in three or more categories of antimicrobials. For Gram-negative bacteria, these categories include extended-spectrum penicillins, carbapenems, cephalosporins, aminoglycosides, and fluoroquinolones; for Gram-positive bacteria, they include ampicillin, vancomycin, fluoroquinolones, fosfomycin, and linezolid. Extended-spectrum beta-lactamases (ESBLs) are a resistance mechanism that is becoming increasingly prevalent among these bacteria. ESBLs are a type of beta-lactamase enzyme that hydrolyze a broader range of beta-lactam antibiotics, including penicillins, many cephalosporins, and aztreonam, thereby compromising the efficacy of most beta-lactams, although they do not affect carbapenems [21]. To detect ESBL-producing Enterobacterales, isolates that demonstrated reduced sensitivity to antibiotics such as ceftazidime, cefpodoxime, ceftriaxone, ceftazidime, and aztreonam underwent confirmation through the double disc synergy test (DDST). This procedure involved comparing the inhibition zones of a third-generation cephalosporin alone and with clavulanic acid incorporated into the discs. Enhanced cephalosporin activity in the presence of clavulanic acid indicated ESBL production in Gram-negative bacilli, in accordance with the CLSI guidelines. Furthermore, phenotypic confirmation assays were performed on isolates suspected of harboring AmpC β-lactamases. This was particularly noted in cases showing resistance to third-generation cephalosporins, where a confirmatory ESBL-negative test was observed, or when there was intermediate sensitivity or resistance to amoxicillin with clavulanic acid and third-generation cephalosporins.

### 2.4. Statistical Analysis

Continuous variables are reported as medians with interquartile ranges, while categorical variables are presented as frequencies and percentages. Normal distribution was assessed using the Kolmogorov–Smirnov one-sample test. Nonparametric data were analyzed using the two-tailed Mann–Whitney U-test. Qualitative variable comparisons were performed using the chi-square test and Fisher’s exact test. For multivariate analysis, logistic regression was employed, with the significance level (α) set at 0.05 for all tests, which were conducted as two-sided analyses. Statistical analyses were performed using IBM SPSS version 22 for Windows (Armonk, New York, NY, USA).

The Clinical Research Ethics Committee of Doctor Peset University Hospital approved this study (code 85/16, September 2016), and it was conducted in accordance with the STROBE guidelines. Every patient, or their legal representative if the patient was unable to give consent, signed the informed consent form.

## 3. Results

Of the 1198 patients admitted with a UTI during this study period, 462 met the inclusion criteria, survived the hospital stay, and were ultimately included in this study. Of these, 35 (7.6%) had a readmission due to a UTI. The patients in the overall series had a median age of 78 (69–86) years, and 50% were women. Diabetes mellitus, moderate–severe chronic kidney disease, and dementia (35.1%, 30.7%, and 25.5%, respectively) were the most common comorbidities, with no difference between the cases that subsequently developed recurrent UTIs (recurrent UTIs) and the cases that did not subsequently develop recurrent UTIs (non-recurrent UTIs) during this study period (see Table 1). Sepsis or septic shock on admission to the hospital were present in 37% of cases, and they were no differences between cases with recurrent and non-recurrent UTIs (37.1% vs. 36.1%, *p* = 0.141). The thirty-day mortality among survivors of the initial hospitalization episode was 4.3%, with no differences between recurrent and non-recurrent UTIs (2.9% vs. 4.4%, *p* = 0.656). The one-year mortality was 24.5%, with no differences between recurrent and non-recurrent UTIs (22.9% vs. 24.6%, *p* = 0.813). However, those with recurrent UTIs had a longer hospital stay (6 days (5–8) vs. 5 days (3–7), *p* = 0.012).

In the univariate analysis (Table 1), recurrent UTI was related to comorbidities (Charlson Comorbidity Index ≥3: 97.1% vs. 85.9%, *p* = 0.039), use of indwelling urinary catheter (37.1% vs. 18.7%, *p* = 0.009), and healthcare-associated UTI (80% vs. 52%, *p* = 0.001), especially previous hospitalization and previous antimicrobial therapy, 

*Escherichia coli* was the most frequently isolated microorganism in the total of the series (59.6%), being significantly more frequent in the non-recurrent urinary tract infection (UTI) group (35.9% in recurrent UTI vs. 61.5% in non-recurrent, *p* = 0.05). In contrast, *Pseudomonas aeruginosa* was associated with recurrent UTIs (20.5% vs. 5.6%, *p* < 0.001). Table 2 presents the microorganisms isolated. With 49 cases involving polymicrobial infections, a total of 511 microorganisms are reported.

One-third of the microbiological isolates met the MDR criteria. Although MDR was more frequent in the recurrent UTI group, no significant differences were observed (20% vs. 13.6%, *p* = 0.050). Regarding the ESBL-producing bacteria, a total of 65 cases (14.1%) were found, of which 7 cases (20%) were in the recurrent UTI group and 58 cases (13.6%) in the control group, with no significant differences (see Table 1).

In the multivariate analysis, recurrent UTI was related to healthcare-associated UTI (OR 2.8, 95% CI 1.1–6.9) and *Pseudomonas aeruginosa* infection (OR 2.7, 95% CI 1.1–7.2) (see Table 3).

Among the thirty-five cases of recurrent UTI, four of them were polymicrobial. A total of 51.3% of the cases were caused by the same microorganism, with a median time of 4 (2.75–7.75) months, compared to the median time of 6.5 (4–8) months for cases caused by a different microorganism (*p* = 0.296); in the first group, half of the cases were acute recurrent UTIs (<3 months), while in the second group, acute recurrent UTI accounted for only 25% (*p* = 0.503). The most common microorganisms in the cases of recurrent UTI caused by the same microorganism were *E. coli* (25.6%), followed by *P. aeruginosa* (7.7%). In cases of recurrent UTI caused by a different microorganism, the second episode was most frequently caused by *E. coli* (20.5%), followed by *K. pneumoniae* (10.3%), while *P. aeruginosa* was the most frequent microorganism in the first episode (12.8%), followed by *E. coli* (10.3%). The most commonly used antibiotics for recurrent UTIs were carbapenems (37.1%), ceftriaxone (22.8%), amoxicillin–clavulanate (8.6%), fluoroquinolones (8.6%), ceftriaxone and gentamycin (5.7%), piperacillin–tazobactam 2.9%), and other combinations (14.2%). 

## 4. Discussion

This study showed that 7.6% of older patients admitted to a medical ward with a complicated UTI were readmitted to the hospital for a recurrent UTI during a one-year follow-up period. We identified HCA-UTI and *Pseudomonas aeruginosa* UTI as risk factors for recurrent UTI. 

Although other studies have found a high rate of readmissions due to infections in patients with sepsis, this study identified a low rate of readmissions due to UTIs among older adults and did not find any differences in hospital readmission rates for UTIs between patients with and without sepsis. This finding is particularly relevant given that half of the patients had sepsis or septic shock. The small percentage of recurrent UTI observed in this study is likely attributable to the fact that these infections were community-acquired in patients admitted to a medical ward, not to the ICU, and who had not undergone surgical or urinary tract instrumentation procedures during their hospital stay. Our findings are consistent with those described in a multicenter retrospective study, which reported an 8% readmission rate due to UTI within 60 days following a complicated UTI [10].

Given the advanced age of the patients, comorbidity was very high, with 97% of those with recurrent UTI having a Charlson Comorbidity Index ≥ 3. Additionally, indwelling urinary catheters were also significantly more common among patients with recurrent UTIs. However, healthcare-associated UTIs were the only clinical–epidemiological factor significantly associated with recurrent UTIs by multivariate analysis. This finding suggests that high-risk patients for recurrent UTI can be identified early through a simple initial assessment by asking three key questions: Has the patient been hospitalized recently? Have they received antibiotics recently? Do they reside in a nursing home? 

As expected, *Escherichia coli* was the most common microorganism identified, and although it was associated with a lower rate of recurrent UTI by univariate analysis, only *Pseudomonas aeruginosa* was significantly associated with recurrent UTI by multivariate analysis. *P. aeruginosa* UTIs have been associated with urinary catheters, male sex, and healthcare-associated UTIs [22,23,24]. However, to the best of our knowledge, this association with recurrent community-acquired UTIs in older people has not been described previously.

MDR bacteria were responsible for 33.5% of all cases, a rate consistent with that in other studies conducted in similar contexts (34.1% to 36.5%) [25,26]. However, this factor did not demonstrate a significant association with recurrent UTIs. Our study included a high proportion of healthcare-associated infections (54.1%), which, in other studies, have shown MDR bacteria prevalence rates as high as 46% [27]. While some recent studies have reported an association between MDR and recurrent UTI, these have focused on outpatient infections and conducted retrospective analyses based on the urine culture from the recurrence [28,29]. The results of this study reveal a stronger association of recurrence with clinical factors such as healthcare-associated infections, suggesting that patient-specific characteristics and circumstances may play a more prominent role in the recurrence of a UTI requiring hospitalization.

It is noteworthy that the average length of stay was longer among patients who developed recurrent UTIs, with a median increase of 1 day. However, higher mortality was not observed during hospitalizations at 30 days, or at one year. This suggests that recurrent UTIs are not related to the severity of these infections. 

In the present study, half of the recurrent UTIs were caused by the same microorganisms as those in primary UTIs. Although it might initially be assumed that these infections were the recrudescence or relapse of the same infection, the prolonged period up to recurrence, with a median of 3 months, suggests that they were mostly reinfections. As anticipated, the time to recurrence was significantly shorter in cases caused by the same microorganism. Our findings are consistent with those of a previous study in which only one-fifth to half of the hospitalizations for recurrent sepsis were due to the recrudescence or relapse of the same infection [5].

The primary strength of our study lies in its prospective design, which targeted a well-defined population and was specifically aimed at examining potential differences between patients who experienced a subsequent UTI and those who did not. In the context of recurrent infections, real-time data collection is crucial to prevent bias and ensure completeness of the data. This study presents several limitations. First, it was carried out at a single institution, so the findings should be confirmed in other settings. Second, this study included only patients with recurrent UTIs who were hospitalized, and it is likely that the number of recurrences would be higher if episodes of recurrent UTIs that did not require hospitalization were considered. Third, the sample size for recurrent UTIs was limited. Notwithstanding these limitations, this study offers valuable insights that may enhance the management of elderly patients with community-acquired UTIs.

## 5. Conclusions

In summary, elderly patients admitted to the hospital with complicated urinary tract infections have a low long-term risk of recurrent UTIs. However, this risk is higher in patients with healthcare-associated infection criteria and in those with *P. aeruginosa* UTIs. Identifying these risk groups may aid in the early detection of recurrent UTIs.

## Figures and Tables

**Table 1:** Epidemiological and clinical characteristics in hospitalized older patients with recurrent urinary tract infection.

	Total N 462	Recurrent UTI N 35 (7.6%)	Non-Recurrent UTI N 427 (92.4%)	*p*
**Epidemiology**				
Female sex, n (%)	231 (50)	21 (60)	210 (49.2)	0.218
Age (years), median [IQR]	78 (69–86)	80 (74–84)	78 (69–86)	0.348
**Comorbidities**				
Charlson ≥ 3, n (%)	401 (86.8)	34 (97.1)	367 (85.9)	**0.039**
Dementia, n (%)	118 (25.5)	13 (37.1)	105 (24.6)	0.102
Diabetes mellitus, n (%)	162 (35.1)	8 (22.9)	154 (36.1)	0.115
COPD, n (%)	61 (13.2)	3 (8.6)	58 (13.6)	0.400
Moderate–severe CKD, n (%)	142 (30.7)	15 (42.9)	127 (29.8)	0.108
Cancer, n (%)	96 (20.8)	10 (28.6)	86 (20.1)	0.237
Indwelling urinary catheter, n (%)	93 (20.1)	13 (37.1)	80 (18.7)	**0.009**
HCA-UTI, n (%)	250 (54.1)	28 (80)	222 (52)	**0.001**
Previous hospitalization, n (%)	138 (29.9)	21 (60)	117 (27.4)	**<0.001**
Previous antimicrobial therapy, n (%)	218 (47.2)	27 (77.1)	191 (44.7)	**<0.001**
Nursing home residence, n (%)	24 (5.2)	1 (2.9)	23 (5.4)	0.517
**Clinical characteristics**				
APN, n (%)	314 (68)	22 (62.9)	292 (68.4)	0.501
Fever, n (%)	360 (77.9)	27 (77.1)	333 (78)	0.908
Acute urinary retention, n (%)	75 (16.2)	6 (17.1)	69 (16.2)	0.879
Barthel <40, n (%)	146 (31.6)	16 (45.7)	130 (30.4)	0.062
APACHE II, median [IQR]	10 (8–14)	12 (9–16)	10 (8–14)	0.064
Sepsis (SOFA ≥ 2) and septic shock, n (%)	171 (37)	17 (48.6)	154 (36.1)	0.141
**Laboratory parameters**				
Lactate ≥2 mg/dL	153 (33.1)	13 (37.1)	140 (32.8)	0.880
Albumin, g/dL	3.4 (3–3.7)	3.2 (2.9–3.6)	3.4 (3–3.7)	0.157
Leukocytosis, median [IQR] cel/mm^3^	13,100 (9400–17,800)	13,300 (9300–17,700)	13,100 (9400–17,900)	0.933
Positive BC/extracted BC (%)	92/255 (36)	8/19 (42.1)	84/236 (35.6)	0.650
Polymicrobial UTI, n (%)	49 (10.6)	4 (10.2)	44 (10.3)	0.462
MDR, n (%)	155 (33.5)	17 (48.6)	138 (32.3)	0.050
ESBL, n (%)	65 (14.1)	7 (20)	58 (13.6)	0.294
IEAT, n (%)	111 (24)	10 (29.4)	101 (23.7)	0.450
**Prognostic characteristics**				
Hospital stay, median [IQR] days	5 (3–7)	6 (5–8)	5 (3–7)	**0.006**
30-day mortality, n (%)	20 (4.3)	1 (2.9)	19 (4.4)	0.656
1-year mortality, n (%)	113 (24.5)	8 (22.9)	105 (24.6)	0.813

COPD, chronic obstructive pulmonary disease; CKD, chronic kidney disease; HCA-UTI, healthcare-associated UTI; APN, acute pyelonephritis; MDR, multidrug-resistant bacteria; ESBLs, extended-spectrum beta-lactamases; IEAT, inadequate empirical antimicrobial therapy. *p* < 0.05 is considered statistically significant (in bold).

**Table 2:** Microbiological isolates in urine culture in hospitalized older patients with recurrent and non-recurrent UTI.

Microorganisms, n (%)	Total N = 511	Recurrent UTI N = 39	Non-Recurrent UTI N = 472	*p*
*Escherichia coli*	310 (60.7)	14 (35.9)	296 (62.7)	**0.004**
*Klebsiella* spp.	76 (14.9)	7 (17.9)	69 (14.6)	0.896
*Proteus mirabilis*	19 (3.7)	1 (2.6)	18 (3.8)	0.741
*Pseudomonas aeruginosa*	33 (6.5)	8 (20.5)	25 (5.3)	**<0.001**
*Enterococcus* spp.	43 (8.4)	5 (12.8)	38 (8.1)	0.574
Other microorganisms	30 (5.9)	4 (10.3)	26 (5.5)	0.223

*p* < 0.05 is considered statistically significant (in bold).

**Table 3:** Multivariate analysis of risk factors for recurrent UTI requiring admission in hospitalized older patients with UTI.

	Univariate Analysis		Multivariate Analysis	
	OR (95%CI)	*p*	OR (95%CI)	*p*
Charlson ≥ 3	5.1 (1.7–37.1)	0.039	3.5 (0.5–26.8)	0.226
Indwelling catheter	2.3 (1.2–4.5)	0.009	1.2 (0.5–2.8)	0.623
Healthcare-associated UTI	3.4 (1.5–7.4)	0.001	2.6 (1.1–6.2)	**0.036**
*Escherichia coli*	0.4 (0.2–0.8)	0.004	0.6 (0.3–1.4)	0.241
*Pseudomonas aeruginosa*	3.4 (2.1–12.1)	<0.001	2.7 (1.1–7.2)	**0.048**

*p* < 0.05 is considered statistically significant (in bold).

## Data Availability

The original contributions presented in the study are included in the article, further inquiries can be directed to the corresponding author.

## References

[1] Chang D.W., Tseng C.-H., Shapiro M.F. (2015). Rehospitalizations Following Sepsis. Crit. Care Med..

[2] Gadre S.K., Shah M., Mireles-Cabodevila E., Patel B., Duggal A. (2019). Epidemiology and Predictors of 30-Day Readmission in Patients with Sepsis. Chest.

[3] Goodwin A.J., Rice D.A., Simpson K.N., Ford D.W. (2015). Frequency, Cost, and Risk Factors of Readmissions Among Severe Sepsis Survivors. Crit. Care Med..

[4] Shankar-Hari M., Saha R., Wilson J., Prescott H.C., Harrison D., Rowan K., Rubenfeld G.D., Adhikari N.K.J. (2020). Rate and Risk Factors for Rehospitalisation in Sepsis Survivors: Systematic Review and Meta-Analysis. Intensive Care Med..

[5] DeMerle K.M., Royer S.C., Mikkelsen M.E., Prescott H.C. (2017). Readmissions for Recurrent Sepsis: New or Relapsed Infection?. Crit. Care Med..

[6] Peng D., Li X., Liu P., Luo M., Chen S., Su K., Zhang Z., He Q., Qiu J., Li Y. (2018). Epidemiology of Pathogens and Antimicrobial Resistanceof Catheter-Associated Urinary Tract Infections in Intensivecare Units: A Systematic Review and Meta-Analysis. Am. J. Infect. Control.

[7] Foxman B., Gillespie B., Koopman J., Zhang L., Palin K., Tallman P., Marsh J.V., Spear S., Sobel J.D., Marty M.J. (2000). Risk Factors for Second Urinary Tract Infection among College Women. Am. J. Epidemiol..

[8] Ikahelmo R., Siitonen A., Heiskanen T., Karkkainen U., Kuosmanen P., Lipponen P., Makela P.H. (1996). Recurrence of Urinary Tract Infection in a Primary Care Setting: Analysis of a I-Year Follow-up of 179 Women. Clin. Infect. Dis..

[9] Foxman B., Barlow R., D’Arcy H., Gillespie B., Sobel J.D. (2000). Urinary Tract Infection: Self-Reported Incidence and Associated Costs. Ann. Epidemiol..

[10] Babich T., Eliakim-Raz N., Turjeman A., Pujol M., Carratalà J., Shaw E., Gomila Grange A., Vuong C., Addy I., Wiegand I. (2021). Risk Factors for Hospital Readmission Following Complicated Urinary Tract Infection. Sci. Rep..

[11] Nicolle L.E. (2016). Urinary Tract Infections in the Older Adult. Clin. Geriatr. Med..

[12] Soh J.G.S., Wong W.P., Mukhopadhyay A., Quek S.C., Tai B.C. (2020). Predictors of 30-Day Unplanned Hospital Readmission among Adult Patients with Diabetes Mellitus: A Systematic Review with Meta-Analysis. BMJ Open Diabetes Res. Care.

[13] Pallett A., Hand K. (2010). Complicated Urinary Tract Infections: Practical Solutions for the Treatment of Multiresistant Gram-Negative Bacteria. J. Antimicrob. Chemother..

[14] Nicolle L.E., Gupta K., Bradley S.F., Colgan R., DeMuri G.P., Drekonja D., Eckert L.O., Geerlings S.E., Köves B., Hooton T.M. (2019). Clinical Practice Guideline for the Management of Asymptomatic Bacteriuria: 2019 Update by the Infectious Diseases Society of America. Clin. Infect. Dis..

[15] Artero A., Ian L., Alberola J., Resa E., Piles L., Madrazo M. (2023). Influence of Sepsis on the Middle-Term Outcomes for Urinary Tract Infections in Elderly People. Microorganisms.

[16] Charlson M.E., Pompei P., Ales K.L., MacKenzie C.R. (1987). A New Method of Classifying Prognostic Comorbidity in Longitudinal Studies: Development and Validation. J. Chronic Dis..

[17] Seymour C.W., Liu V.X., Iwashyna T.J., Brunkhorst F.M., Rea T.D., Scherag A., Rubenfeld G., Kahn J.M., Shankar-Hari M., Singer M. (2016). Assessment of Clinical Criteria for Sepsis for the Third International Consensus Definitions for Sepsis and Septic Shock (Sepsis-3). JAMA—J. Am. Med. Assoc..

[18] Singer M., Deutschman C.S., Seymour C., Shankar-Hari M., Annane D., Bauer M., Bellomo R., Bernard G.R., Chiche J.D., Coopersmith C.M. (2016). The Third International Consensus Definitions for Sepsis and Septic Shock (Sepsis-3). JAMA—J. Am. Med. Assoc..

[19] Leone M., Bourgoin A., Cambon S., Dubuc M., Albanèse J., Martin C. (2003). Empirical Antimicrobial Therapy of Septic Shock Patients: Adequacy and Impact on the Outcome. Crit. Care Med..

[20] Magiorakos A.P., Srinivasan A., Carey R.B., Carmeli Y., Falagas M.E., Giske C.G., Harbarth S., Hindler J.F., Kahlmeter G., Olsson-Liljequist B. (2012). Multidrug-Resistant, Extensively Drug-Resistant and Pandrug-Resistant Bacteria: An International Expert Proposal for Interim Standard Definitions for Acquired Resistance. Clin. Microbiol. Infect..

[21] Clifford K.M., Grelle J.L., Tawwater J.C., Ramanathan M., Duncan N. (2019). Management of Extended-Spectrum Beta-Lactamase Urinary Tract Infections: Diagnostic and Treatment Considerations for the Older Adult. Sr. Care Pharm..

[22] Weiner L.M., Webb A.K., Limbago B., Dudeck M.A., Patel J., Kallen A.J., Edwards J.R., Sievert D.M. (2016). Antimicrobial-Resistant Pathogens Associated with Healthcare-Associated Infections: Summary of Data Reported to the National Healthcare Safety Network at the Centers for Disease Control and Prevention, 2011–2014. Infect. Control Hosp. Epidemiol..

[23] Esparcia A., Madrazo M., Alberola J., López-Cruz I., Eiros J.M., Nogueira J.M., Artero A. (2019). Community-Onset Pseudomonas Aeruginosa Urinary Sepsis in Elderly People: Predictive Factors, Adequacy of Empirical Therapy and Outcomes. Int. J. Clin. Pract..

[24] Mittal R., Aggarwal S., Sharma S., Chhibber S., Harjai K. (2009). Urinary Tract Infections Caused by Pseudomonas Aeruginosa: A Minireview. J. Infect. Public Health.

[25] Gomila A., Carratalà J., Eliakim-Raz N., Shaw E., Tebé C., Wolkewitz M., Wiegand I., Grier S., Vank C., Cuperus N. (2019). Clinical Outcomes of Hospitalised Patients with Catheter-Associated Urinary Tract Infection in Countries with a High Rate of Multidrug-Resistance: The COMBACTE-MAGNET RESCUING Study. Antimicrob. Resist. Infect. Control.

[26] Bischoff S., Walter T., Gerigk M., Ebert M., Vogelmann R. (2018). Empiric Antibiotic Therapy in Urinary Tract Infection in Patients with Risk Factors for Antibiotic Resistance in a German Emergency Department. BMC Infect. Dis..

[27] Karve S., Ryan K., Peeters P., Baelen E., Rojas-Farreras S., Potter D., Rodríguez-Baño J. (2018). The Impact of Initial Antibiotic Treatment Failure: Real-World Insights in Patients with Complicated Urinary Tract Infection. J. Infect..

[28] Fromer D.L., Cheng W.Y., Gao C., Mahendran M., Hilts A., Duh M.S., Joshi A.V., Mulgirigama A., Mitrani-Gold F.S. (2024). Likelihood of Antimicrobial Resistance in Urinary E Coli Isolates among US Female Patients with Recurrent vs Non-Recurrent Uncomplicated Urinary Tract Infection. Urology.

[29] Gavilanes-Parra S., Chavero-Guerra P., Hernández-Castro R., Villanueva-Recillas S., Manjarrez-Hernández A. (2024). Antimicrobial Resistance in Uropathogenic Escherichia Coli Strains Isolated from Relapses from Recurrent Urinary Tract Infections. Microb. Drug Resist..

